# The first step in the development of text mining technology for cancer risk assessment: identifying and organizing scientific evidence in risk assessment literature

**DOI:** 10.1186/1471-2105-10-303

**Published:** 2009-09-22

**Authors:** Anna Korhonen, Ilona Silins, Lin Sun, Ulla Stenius

**Affiliations:** 1 Computer Laboratory, University of Cambridge, 15 JJ Thomson Avenue, Cambridge CB3 0FD, UK; 2 Institute of Environmental Medicine, Karolinska Institutet, S-17177, Stockholm, Sweden

## Abstract

**Background:**

One of the most neglected areas of biomedical Text Mining (TM) is the development of systems based on carefully assessed user needs. We have recently investigated the user needs of an important task yet to be tackled by TM -- Cancer Risk Assessment (CRA). Here we take the first step towards the development of TM technology for the task: identifying and organizing the scientific evidence required for CRA in a taxonomy which is capable of supporting extensive data gathering from biomedical literature.

**Results:**

The taxonomy is based on expert annotation of 1297 abstracts downloaded from relevant PubMed journals. It classifies 1742 unique keywords found in the corpus to 48 classes which specify core evidence required for CRA. We report promising results with inter-annotator agreement tests and automatic classification of PubMed abstracts to taxonomy classes. A simple user test is also reported in a near real-world CRA scenario which demonstrates along with other evaluation that the resources we have built are well-defined, accurate, and applicable in practice.

**Conclusion:**

We present our annotation guidelines and a tool which we have designed for expert annotation of PubMed abstracts. A corpus annotated for keywords and document relevance is also presented, along with the taxonomy which organizes the keywords into classes defining core evidence for CRA. As demonstrated by the evaluation, the materials we have constructed provide a good basis for classification of CRA literature along multiple dimensions. They can support current manual CRA as well as facilitate the development of an approach based on TM. We discuss extending the taxonomy further via manual and machine learning approaches and the subsequent steps required to develop TM technology for the needs of CRA.

## Background

Biomedical Text Mining (TM) has become increasingly popular due to the great need to provide access to the tremendous body of texts available in biomedical sciences. Considerable progress has been made in the development of basic resources (e.g. ontologies, annotated corpora) and techniques in this area, e.g. in Information Retrieval (IR) (i.e. identification of relevant documents) and Information Extraction (IE) (i.e. identification of specific information in the documents, e.g. proteins and genes, and specific relations between them), and research has began to focus on increasingly challenging tasks, e.g. summarization and the discovery of novel information in biomedical literature [1-4].

The major current challenge is to extend TM techniques with richer and deeper analysis and to apply them to support real-world tasks in biomedicine. In recent past, there has been an increasing trend towards research which is driven by actual user needs rather than by technical developments [5]. Corpus annotation and classification schemes applicable to a wider variety of biomedical literature have been developed to support biologists with diverse TM needs [6,7]. Shared tasks (e.g. BioCreative and the TREC Genomics track) targeting the actual workflow of biomedical researchers have appeared, along with studies exploring the TM needs of specific tasks (e.g. literature curation, library services for biomedical applications) [8,9]. Several practical tools have been developed for the use of working scientists which can support IR and IE from biomedical literature [10-13]. However, the understanding of user needs is still one of the neglected areas of biomedical TM, and further user-centered evaluations and systems grounded in real-life tasks are required to determine which tools and services are actually useful [14].

In our recent work, we investigated the user needs of a challenging task yet to be tackled by text mining: Cancer Risk Assessment (CRA) [15,16]. CRA is a task which involves examining existing published evidence to determine the relationship between exposure to a chemical and the likelihood of developing cancer from that exposure [17]. It has become increasingly important over the past years as the link between environmental chemicals and cancer has become evident and tight legislations governing chemical safety have been introduced worldwide. For example, the recently established European Community REACH (Registration, Evaluation, Authorisation and Restriction of Chemical substances) legislation requires that all the chemicals manufactured or imported in a high quantity must undergo thorough CRA (EC 1907/2006) [18].

Performed manually by experts in health related institutions, CRA is a demanding exercise which requires combining scientific knowledge with elaborate literature review. It involves searching, locating and interpreting relevant information in repositories of scientific peer reviewed journal articles - a process which can be extremely time-consuming because the data required for CRA of just a single carcinogen may be scattered across thousands of articles. Over the recent years, while the need for CRA has grown, the task has also turned increasingly complex due to the rapid development of molecular biology techniques, the increased knowledge of mechanisms involved in cancer development, and the exponentially growing volume of CRA literature (e.g. the MEDLINE database [19] of biomedical research articles expanded with over 0.5 M references last year and now includes over 17 million in total). Under these circumstances, CRA is getting too challenging to manage via manual means.

To gain an understanding of how TM could best assist CRA, we conducted an initial study where we interviewed 14 experienced risk assessors working for different national and international CRA authorities in Sweden^1 ^[16]. During this study, the risk assessors described the following steps of their work: (1) identifying the journal articles relevant for CRA of the chemical in question, (2) identifying the scientific evidence in these articles which help to determine whether/how the chemical causes cancer, (3) classifying and analysing the resulting (partly conflicting) evidence to build the toxicological profile for the chemical, and (4) preparing the risk assessment report. These steps are conducted largely manually, relying on standard literature search engines (e.g. provided with PubMed) and word processors as technical support. CRA of a single chemical may take several years when done on a part time basis. The risk assessors were unanimous about the need to increase the productivity of their work to meet the current CRA demand. They reported that locating and classifying the scientific evidence in literature is the most time consuming phase of their work and that a tool capable of assisting this phase and ensuring that all the potentially relevant evidence is found would be particularly helpful.

It became clear to us that a prerequisite for the development of such a tool would need to be an extensive specification of the scientific evidence used for CRA. This evidence -- which forms the basis of all the subsequent steps of CRA -- is described in the guideline documents of major international CRA agencies, e.g. European Chemicals Agency [20] (ECHA), the United States Environmental Protection Agency [17] (EPA), and the International Agency for Research on Cancer (IARC) [21]. The guideline documents describe various human, animal (*in vivo*), cellular (*in vitro*) and other mechanistic data which provide evidence for both hazard identification (i.e. the assessment of whether a chemical is capable of causing cancer) and the assessment of the Mode of Action (MOA) (i.e. the sequence of key events that result in cancer formation, e.g. mutagenesis, increased cell proliferation, and receptor activation). However, our investigation showed that although these documents constitute the main reference material available for CRA, they cover the main types of evidence only, do not specify the evidence at the level of detail required for comprehensive data gathering (e.g. do not provide complete lists of relevant keywords or terms) and are not updated regularly to include the latest developments in biomedical sciences. For example, the most recent EPA CRA guideline was published in 2005 and the data requirements have not been updated since then.

The same guidelines emphasise, however, the importance of investigating all the published scientific data on the chemical in question which might be of potential relevance for CRA. For example, according to ECHA [20] "failure to collect all of the available information on a substance may lead to duplicate work, wasted time, increased costs and potentially unnecessary animal use" (page 7). Recent research has revealed that conflicting risk assessments of the same chemical are surprisingly common [22,23]. Inadequate or imbalanced data may give rise to such problems. Extensive data gathering is therefore essential not only for the coverage but also for the accuracy of CRA.

Where the guidelines fail to provide sufficient information, risk assessors rely on their experience and expert knowledge. This is not ideal since chemical carcinogenesis is such a complex process that even the most experienced risk assessor is incapable of memorizing the wide range of relevant evidence without the support of a thorough specification.

Here we report the work we did on obtaining a more adequate specification of the scientific evidence for CRA. Ideally, a comprehensive knowledge resource is needed which specifies the range of relevant evidence and provides extensive lists of keywords to support the gathering of this evidence in literature. Given the dynamic nature of CRA data, the best approach long term would be to develop technology for automatic acquisition and updating of such a resource from CRA literature [1,2]. However, the very development of such technology requires target specification of the scientific evidence more comprehensive than that currently provided. Therefore, in this first work, we opted for expert annotation of biomedical literature according to the evidence it offers for CRA.

Following the recommended practices of biomedical corpus design by Cohen *et al*. [24] as far as practical, we constructed a representative, balanced CRA corpus of 1297 MEDLINE abstracts from a set of journals typically used for CRA. A user-friendly annotation tool was designed which experts could use to annotate abstracts (i) for the relevance for CRA and (ii) according to the types of evidence they provide for the task. Three experts (experienced risk assessors) agreed on the annotation guidelines and produced a corpus which contains 1164 abstracts judged as relevant and annotated for 1742 unique keywords (words or phrases) indicating the evidence they offer for CRA. The experts grouped the keywords according to the types of evidence they provide for the task, and organized them into a taxonomy which contains 48 distinct classes and covers a variety of data related to carcinogenic activity, MOA and toxicokinetics. We measure the inter-annotator agreement of both relevance and keyword annotation tasks. In addition, we report a series of experiments which involve training and testing automatic classifiers to assign PubMed abstracts to taxonomy classes. Finally, a simple user test in a near real-world CRA scenario is reported. The evaluation we report demonstrates that our taxonomy is highly accurate and can be useful for practical CRA. The materials we have produced can thus provide valuable support for manual CRA as well as facilitate the development of an approach based on TM. We discuss refining and extending the taxonomy further via manual and machine learning approaches, and the subsequent steps required to develop TM to support the entire CRA workflow.

The rest of this paper is organized as follows: The Methods section introduces the CRA corpus, the annotation tool, the annotation guidelines, the principles of taxonomy construction, and the automatic classification methods. The Results section describes first the annotation work and the resulting taxonomy. The results of the inter-annotator agreement tests, the automatic classification experiments and the user-test are then reported. The Discussion and Conclusion section concludes the paper with comparison to related research and directions for future work.

## Methods

### Cancer Risk Assessment Taxonomy

Three experienced risk assessors helped to construct the resources described in the following four sections, respectively: (i) a representative corpus of CRA literature for parts of hazard identification (i.e. the assessment of whether a chemical is capable of causing cancer), (ii) a tool for expert annotation of the corpus, (iii) an annotated corpus, and (iv) a taxonomy which classifies and organizes the scientific evidence discovered in the corpus.

#### CRA corpus

Most CRA literature is now available electronically via online resources such as the National Library of Medicine's PubMed system [25] and databases such as the Integrated Risk Information System (IRIS) [26], TOXicology Data NETwork [27] and the Organisation for Economic Co-operation and Development (OECD) Global Portal to Information on Chemical Substances [28]. As PubMed is by far the most frequently used resource in CRA, we selected 15 journals available via this system which are used frequently for CRA and jointly provide a good coverage of the main types of scientific evidence relevant for the task. From these 15 journals (listed in Table 1), all the abstracts from years 1998 to 2008 which include one of eight test chemicals were downloaded for further analysis. The eight chemicals are shown in Table 2. They were selected by the experts on the basis that they are (i) well-researched using a range of scientific tests and (ii) represent the two most common MOAs - *genotoxic *and *non-genotoxic*^2^. Although full articles are known to provide richer data for TM purposes than abstracts, [24], we focussed on abstracts in this work because they are the typical starting point in CRA. The literature search was limited to recent 10 years. Since many of the selected chemicals are well-studied, this yielded a sufficient number of abstracts for annotation. All the retrieved abstracts were included, except for benzo(a)pyrene for which only the latest 200 (out of the c. 900 in total) were considered. The resulting corpus of 1297 abstracts is distributed per chemical as shown in Table 3.

**Table 1:** Selected journals

**Journal name**	**Number of abstracts**
Archives of Toxicology	56
Cancer Letters	80
Cancer Research	75
Carcinogenesis	135
Chemical Research in Toxicology	106
Chemico-Biological Interaction	169
Environmental and Molecular Mutagenesis	45
Environmental Health Perspectives	97
Mutagenesis	31
Mutation research	142
Regulatory Toxicology and Pharmacology	24
Science of the Total Environment	30
Toxicological Sciences	164
Toxicology and Applied Pharmacology	106
Toxicology Letters	110

**Table 2:** Selected chemicals

**Chemical**	**MOA**	**Occurrence**	**Causes**	**Examples of tumors**
1,3-Butadiene	Genotoxic	Used in production of synthetic rubber.	Mutations	Leukemia
Benzo(a)pyrene	Genotoxic	Incomplete burning of coal, oil and garbage.	Mutations	Skin, lung
Diethylnitrosamine	Genotoxic	Found in foods, tobacco products and industrial solvents.	Mutations	Liver
Styrene	Genotoxic	Used in the manufacture of plastics and rubber.	Mutations	Lung
Chloroform	Non-genotoxic	Laboratory solvent and dry cleaning agent.	Cell death, regenerative proliferation. Hormonal receptor activation.	Liver, kidney
Diethylstilbestrol	Non-genotoxic	Synthetic estrogen.		Vagina, breast
Fumonisin B1	Non-genotoxic	A toxin produced by Fusarium moulds, found in foods.	Cell death, regenerative proliferation.	Oesophageal cancer, liver
Phenobarbital	Non-genotoxic	Barbiturate used as anticonvulsant.	Stimulates proliferation inhibits apoptosis.	Liver (in laboratory animals)

**Table 3:** Number of abstracts per chemical

**Chemical name**	**Number of abstracts**
1,3-Butadiene	195
Benzo(a)pyrene	200
Chloroform	96
Diethylnitrosamine	221
Diethylstilbestrol	145
Fumonisin B1	80
Phenobarbital	270
Styrene	162

#### Annotation tool

Risk assessors typically (i) read each abstract retrieved by PubMed to determine its relevance for CRA, and (ii) classify each relevant abstract based on the types of evidence it provides for CRA. We designed a tool for expert annotation which imitates this process as closely as possible.

The tool provides two types of functionality. The first enables the experts to classify abstracts using the classical IR concept of Document Relevance. The judgements are made at the document level. An abstract is marked as relevant or irrelevant if the expert deems after reading the title and the abstract that it is not relevant for CRA. An abstract can also be marked as unsure. The second functionality enables the experts to annotate such keywords (words and phrases) in abstracts and their titles which indicate scientific evidence relevant for examining the carcinogenic properties of chemicals. This annotation is grounded in actual pieces of text. Initially a very shallow taxonomy (including only general scientific evidence pertaining to human, animal and cellular data) and two types of MOA (genotoxic and non-genotoxic) was integrated inside the tool. As explained below, this was gradually extended as the annotation progressed further. The tool permits annotating any number of relevant keywords in the abstracts, attaching them to any (leaf or internal) node in the taxonomy, and classifying the same text in more than one way.

**Figure 1:**
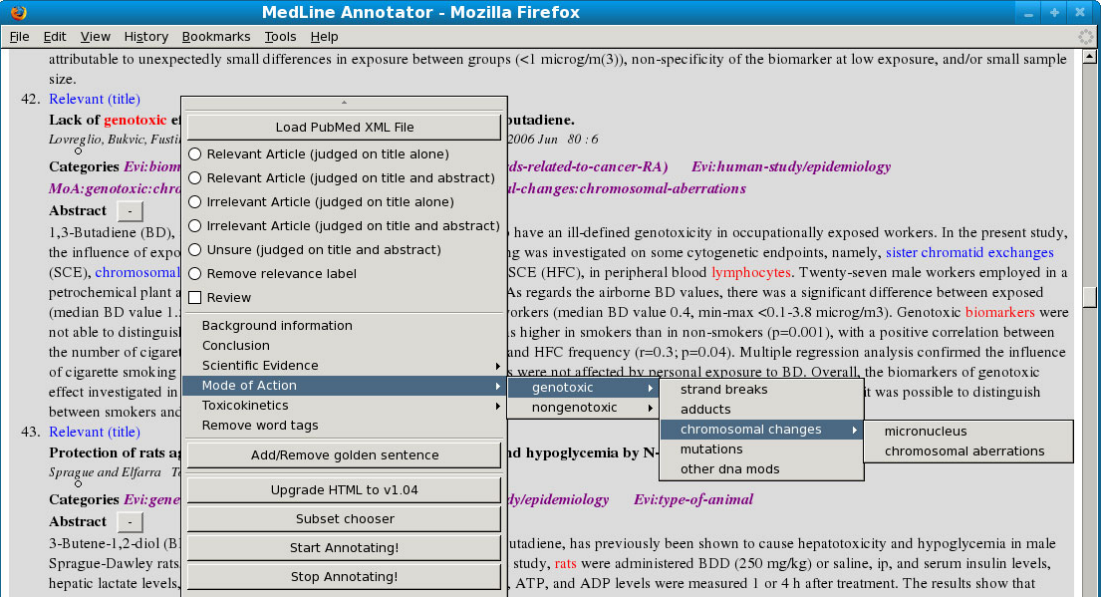
**Annotation tool: This figure displays the annotation tool**.

The tool was implemented inside the Mozilla Firefox browser using its extension facility. The implementation enables PubMed abstracts to be viewed inside a familiar web-browsing environment and also to be classified according to the specialized taxonomy. Previous work has observed that integrating custom functions within a familiar document browsing environment greatly encourages user uptake [8]. The CRA analysed abstracts could then be stored, reviewed by others and edited. In this way, the deployment of the analysis in a genuine CRA scenario was able to be quickly tested. A screenshot illustrating the annotation tool is provided in Figure 1.

#### Annotation guidelines

The three experts agreed on the guidelines of document relevance and keyword annotation. The guidelines were developed so that one of the experts conducted annotation based on the initial set of principles agreed among the three experts. The other two evaluated the outcome, the disagreement cases were discussed, and the annotation principles were improved where possible. This process (crucial for maintaining quality) was repeated on a subset of the corpus for several times. The guidelines described below are the final result of this work.

##### Relevance annotation

The aim of the relevance annotation is to classify each abstract in the corpus as relevant, irrelevant or unsure with regard to hazard identification and MOA assessment. Since current CRA guidelines do not provide clear advice for this step, the experts agreed on the annotation principles based on their experience with CRA literature. They agreed that the abstracts of articles focussed on initiator/promoter studies or studies on chemoprevention where the chemical in question is used as a model compound should be considered as relevant because they invariably contain data interesting for CRA. They also agreed that the abstracts of articles focussed only on exposure assessment should not be included because they are not suitable for hazard identification. Any other abstracts should be examined carefully for their relevance for CRA using expert judgement.

As a general rule, when an abstract contains evidence relevant for CRA, it should be classified as relevant, even when the name of the chemical of interest is missing in the abstract or the title. Although some such abstracts may turn out to be irrelevant after reading the full article, experts agreed that since they are potentially relevant, they should be included for further assessment as not to lose data valuable for CRA. Appendix 1 shows sentences from some abstracts judged as relevant where the evidence for relevance is highlighted using bold font.

When the abstract (or its title) either contains no evidence for relevance or contains "negative" evidence (evidence which indicates irrelevance), it is classified as irrelevant. Negative evidence includes, for example, diseases or endpoints unrelated to cancer (e.g. effects on muscle contraction or loss of hearing). Appendix 2 shows example sentences from irrelevant abstracts with negative evidence highlighted.

Not all the abstracts are clearly relevant or irrelevant according to these criteria. Some abstracts include only vague evidence for relevance. Some contain conflicting (both positive and negative) evidence. For example, effects on cell death (relevant for CRA) may occur in the same abstract with plants (irrelevant for CRA). Others include evidence whose association with cancer development is currently unclear or evidence which focuses on studies on single chemicals in complex mixtures such as tobacco smoke. The experts decided to deal with such challenging abstracts on case by case basis.

##### Keyword annotation

The aim of the keyword annotation is to highlight such keywords (words or phrases) in abstracts which indicate relevant evidence for hazard identification and MOA assessment of chemicals. The experts found keyword annotation easy to understand since they typically look for words and phrases while reading abstracts, and since existing CRA guidelines provide some sample keyword lists to support data gathering. It was agreed that the keyword annotation should focus on the following main types of data considered in CRA [17]:

**1. Carcinogenic activity**. Various types of human, animal (in vivo), cellular (in vitro) and other mechanistic data provide evidence for CRA. Risk assessors pay attention to a variety of keywords indicating different study types in texts when aiming to locate this data. For example, the appearance of the keyword *haemoglobin-adduct *may indicate that the abstract focuses on a biomarker study, and the appearance of *humans *in the same abstract may suggest that the biomarker study focuses on humans rather than e.g. animals. It was agreed that the experts would annotate all the keywords which jointly identify the types of scientific data offered by the abstract.

**2. Mode of Action (MOA)**. A MOA is a core concept in CRA. It specifies the key events leading to cancer, explaining the genetic and cellular alterations which result in the appearance of the scientific data mentioned above. Different chemicals act according to different MOAs, and risk assessors search evidence for a specific MOA type in abstracts. Two main types of MOA can be distinguished: genotoxic and non-genotoxic (both can be further divided into various subtypes). Chemicals acting by a genotoxic MOA interact with DNA, while chemicals acting by a non-genotoxic MOA induce cancer without interfering directly with DNA. To identify the MOA type in question, risk assessors examine scientific data (e.g. haemoglobin-adduct data) in conjunction with mutations in genes. A number of genes (e.g. oncogenes and tumor suppressor genes) are known to be involved in cancer development and are therefore used as evidence. Some of these are shown in Table 4, together with proteins regulated by them. For example, an abstract which reports mutation in p53 gene resulting in decreased expression of its downstream protein p21 suggests genotoxic MOA while an abstract which describes activation of FasL resulting in Caspase-8 mediated apoptosis suggests non-genotoxic MOA.

**Table 4:** Examples of cancer related genes and proteins regulated by these genes

**Genes**	**Regulated proteins**
p53	Noxa, Puma, p21, Mdm2
PTEN	PIP3, Akt, Cyclin D1
Ras	Raf1, Mek, Erk, Akt
FasL	Caspase-8, Caspase-3, Bid
RB	E2F, Cyclin E, Cyclin A

**3. Toxicokinetics**. Toxicokinetics describes the process of uptake of chemicals by the body: the metabolism and biotransformation, and the distribution and excretion. Accurate MOA classification of some chemicals requires evidence for a certain type of toxicokinetics. For example, aflatoxinB1 needs to be activated by CYP 450 to be able to bind to DNA.

Many abstracts focus on several chemicals and/or refer to results conducted in previously published experiments. It was agreed that the experts would focus only on the chemical of interest and on new rather than previously published results. For maximum accuracy, the experts were not required to annotate every potentially relevant keyword but only the ones which they perceived as the most important or dominant. Where the same keyword appeared several times in the abstract or appeared in different forms (e.g. *tumors*, *tumor*) it was annotated at least once.

#### Principles of taxonomy creation

The keyword annotation resulted in lists of words and phrases indicating evidence for CRA. The next task was to classify this evidence and organize it into a taxonomy. We mentioned earlier that initially only a very shallow taxonomy was implemented inside the annotation tool. As the keyword annotation progressed, this taxonomy was gradually extended and refined further with novel classes and class members. The resulting taxonomy relies solely on expert knowledge. Experts were merely advised on the main principles of taxonomy creation: the classes should be conceptually coherent and their hierarchical organization should be in terms of coherent sub- and superordinate relations.

### Automatic Classification

To find out whether the classification created by experts provides a good representation of the corpus data and is machine learnable, we conducted a series of abstract classification experiments. A number of standard feature extraction, feature selection and machine learning methods were used and compared in these first experiments to identify optimal methodology for our data and task. These are introduced in the subsequent sections.

#### Feature extraction

The first step of our text categorization (TC) approach is to transform documents into a feature vector representation. We experimented with two document representation techniques. The first one is the simple 'bag of words' approach (*BOW*) which considers each word in the abstract as a separate feature. *BOW *was evaluated using three methods which have proved useful in previous TC work: (i) stemming (using the Porter stemmer [29]) which removes affixes from words, (ii) the TFIDF weighting [30], and (iii) stop word removal (which removes the uninformative words, e.g. articles and prepositions).

The second technique is the recent 'bag of substrings' (*BOS*) method by [31] which considers the whole abstract as a string and which extracts from it all the length *p *substrings without affix removal. *BOS *has proved promising in recent biomedical TC experiments [31,32] and unlike a traditional grammatical stemmer, it does not require domain tuning for optimal performance. Because it generates substrings with a fixed length *p*, a word shorter than *p *- 2 can get obscured by its context^3^. For example, 'mice' could be transformed to ' _mice_a', '_mice_b',..., which is less informative than the original word form. Therefore, we enriched the *BOS *features with word forms shorter than *p *- 2.

#### Feature selection

We employed two feature selection methods for dimensionality reduction. The first is Information Gain (*IG*) which has proved useful in TC [33]. Given a feature's distribution *X *and class label distribution *Y*, *IG*(*X*) = *H*(*Y*) - *H*(*Y *| *X*), *H*(*X*) is the entropy of *X*. The second method *fscore *optimises the number of features (*N*). Features are first ranked using the simple *fscore *criterion [34], and *N *is selected based on the performance of the SVM classifier using the *N *features.

#### Classification

We experimented with three well-known classifiers: Naive Multinomial Bayesian (*NMB*), Complement Naive Bayesian (*CNB*) [35] and Linear Support Vector Machines (*L-SVM*) [36].

*NMB *is a simple, widely used classifier in TC [30]. It selects the class *C *with the maximum probability given the document *d*: argmax_*c *_*Pr*(*C*) Π_*w*∈ *d *_*Pr*(*X *= *w*|*C*). *Pr*(*C*) can be estimated from the frequency of documents in *C*. *Pr*(*X *= *w*|*C*) is estimated as the fraction of tokens in documents of class *C *that contain *w*.

*CNB *extends *NMB *by addressing the problems it has e.g. with imbalanced data and the weight magnitude error. The class *c *of a document is: .  is the number of times term *i *occurs in classes other than *c*. *α *and *α*_*i *_are the smoothing parameters. *p*(*θ*_*c*_) is the prior distribution of class *c*.

We used WEKA software environment [37] for the implementation of *NMB *and *CNB*.

SVMs have been reported to outperform other TC methods on many TM tasks and have the benefit that they work well even when the data is sparse. *L-SVM *is the basic type of SVM which produces a hyperplane that separates two-class samples with the maximum margin. The method handles high dimensional data efficiently, and has been shown to perform well in TC [38]. Given the data set *X *= (**x**_**1**_, *y*_1_),...,(**x**_**n**_, *y*_*n*_) *y*_*i *_∈ {-1, +1}, *L-SVM *requires a solution **w **to the following unconstrained optimisation problem: . Cost parameter *C *was estimated within range 2^2^,...,2^5 ^on training data using cross validation. The *C *of the positive class was weighted by class population ratio . The feature vector was *normalized *before inputting into *L-SVM*, because the scaling is important for *SVM *[39]. LIBLINEAR [40] was used for the implementation of *L-SVM*.

## Results

The following sections report our results first for the annotation and taxonomy construction tasks, then for the automatic classification experiments, and finally for the user test which involves applying the automatic classification technology to corpus data of unseen chemicals and evaluating the resulting classified data using expert judgment.

### Annotation tasks

Using the annotation tool and the guidelines we have described, the experts annotated each of the 1297 abstracts for (i) relevance and (ii) keywords. They classified 89.4% of the abstracts as relevant, 10.1% as irrelevant and 0.5% as unsure. The small proportion of unsure abstracts can be explained by the general CRA principle which the risk assessors followed which encourages them to collect all the data of potential relevance for further assessment [17,20].

We used the widely employed Kappa statistics [41] to measure the level of inter-annotator agreement in relevance classification. Although not a fully ideal measure (see e.g. [42] for a discussion and criticism), we adopted it for this first annotation effort due to its familiarity. We used the Cohen's chance agreement model [41] since Eugenio and Glass [43] have shown that the model is better than Siegel and Castellan's model [44] in the studies such as ours where the distribution of categories is not equal between the annotators.

The Kappa statistics was calculated on data which two experts annotated independently^4^. 26 abstracts per chemical were selected randomly from the 15 and 16 journals listed in Table 1 and Appendix 3, respectively. The 16 journals in Appendix 3 were selected on the basis of their likely irrelevance for CRA. They were included to make the annotation task harder, given the high proportion of relevant abstracts among the 15 journals used for corpus creation. The resulting test data contains 208 abstracts in total. The test set was kept intentionally small to facilitate thorough error analysis. Table 5 shows basic statistics with regard to the experiment: the values that the Kappa function requires as input. The Kappa statistics K is calculated as follows:

**Table 5:** Statistics used in the inter-annotator agreement test

	**Annotator 2 rel**	**Annotator 2 irr**	**Annotator 1 total**
Annotator 1 rel	145(*p*_11_= 0.697)	16(*p*_12 _= 0.077)	161(*p*_1· _= 0.774)

Annotator 1 irr	8 (*p*_21_= 0.038)	39(*p*_22 _= 0.186)	47 (*p*_2· _= 0.226)

Annotator 2 total	153(*p*_·1 _= 0.736)	55(*p*_·2 _= 0.264)	208(1)

(1)

*p*_*a *_measures the observed level of agreement while *p*_*e *_measures the chance agreement between two annotators.

The maximum value of Kappa score occurs when the agreement is one (*p*_*a *_= 1). The minimum value 0 indicates that the agreement is by chance (*p*_*a *_= *p*_*e*_). Our Kappa result is 0.68. According to the scale of [45], this value indicates substantial agreement between the annotators.

The annotators disagreed on 24 (11.5%) of the abstracts. Half of the disagreements (12) were due to one of the annotators failing to notice keywords in text, and thus erroneously judging abstracts as irrelevant. These disagreements do not warrant improving the annotation guidelines but are likely to decrease when the annotators gain more experience. The other half of the disagreements (12) were caused by one (or both) of the following problems:

• Environmental studies can provide important evidence for CRA, but it is unclear what kind of environmental studies should be included in CRA (e.g. measurements of exposure levels in specific cities, areas, or ethnic/occupational groups).

• Conflicting information in abstracts: the abstracts can include both relevant and irrelevant information, making it difficult for the annotators to decide on the relevance.

**Figure 2:**
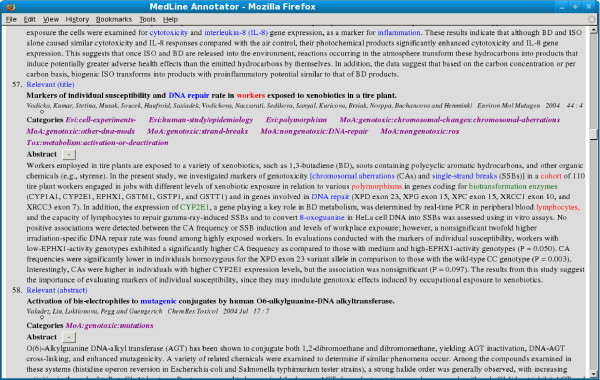
**Annotated abstract: Figure displaying the annotated abstract**.

The keyword annotation was done for the 1164 abstracts deemed relevant during the relevance annotation. Keywords mentioned in existing CRA guideline documents were identified along with many novel ones missing in them (these are illustrated together with the taxonomy in the following section). A total of 1742 unique keywords were identified. Figure 2 shows an example of an annotated abstract where keywords indicating different types of evidence are highlighted in blue, red, and green fonts. Since the experts were not required to annotate every single relevant keyword, calculating inter-annotator agreement was not meaningful. However, we evaluated the keyword annotation as part of the taxonomy classification and provide the corresponding inter-annotator agreement scores in the following section.

### Taxonomy

The keyword annotation of the first two chemicals (one genotoxic and one non-genotoxic) resulted in several updates in the classification and considerable extension of the initial taxonomy implemented inside the annotation tool. During the annotation of the subsequent six chemicals, only minor changes were required: some classes were combined, divided or refined following the discussion among the experts. The resulting taxonomy consists of three sub-taxonomies, corresponding to 1) carcinogenic activity, 2) MOA and 3) toxicokinetics.

#### Carcinogenic activity

**Figure 3:**
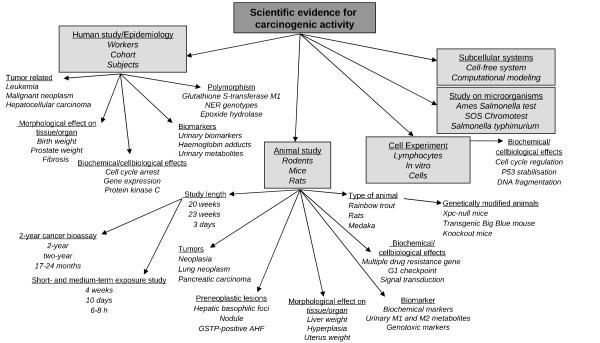
**Taxonomy for carcinogenic activity: A flow chart displaying taxonomy for carcinogenic activity**.

The first sub-taxonomy, shown in Figure 3, specifies the types of data which provide evidence for carcinogenic activity. Five main classes are included: "human study/epidemiology", "animal study", "cell experiments", "study on micro-organisms", and "subcellular systems". The first three of these divide further into subclasses. For example, "human study/epidemiology" has several sub-classes corresponding to different types of human studies: "polymorphism", "biomarkers", "tumor related effects", "morphological effects on tissues and organs", and "biochemical and cellbiological effects". Each class is illustrated in the figure by 2-3 example studies, corresponding to individual keywords in the annotated abstracts (e.g. *cell cycle arrest, gene expression *and *protein kinase C *for "biochemical and cellbiological effects"). Most child nodes in this sub-taxonomy have a type of relation with their parent class. For example, "polymorphism" is here a type of "human study". The only exceptions are "study length" and "the type of animals" under "animal studies" class. They provide additional information about animal studies important for CRA.

#### Mode of Action

**Figure 4:**
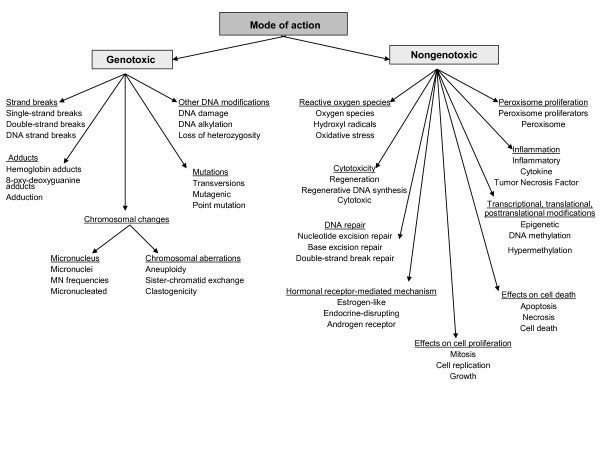
**Taxonomy for mode of action: A flow chart displaying taxonomy for mode of action**.

The second sub-taxonomy focuses on MOA and specifies the types of scientific evidence required for MOA classification. Shown in Figure 4, this sub-taxonomy currently covers the two most frequent MOA types. In this taxonomy, the sub-classes of "genotoxic" and "non-genotoxic" specify different types of evidence for the MOA type in question. For example, "strand breaks", "adducts", "chromosomal changes" ("micronucleus" and "chromosomal aberrations"), "mutations" and "other DNA modifications" each provide different types of evidence for the genotoxic MOA.

#### Toxicokinetics

**Figure 5:**
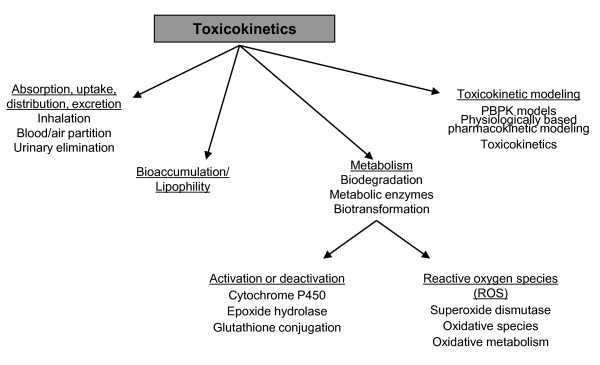
**The toxicokinetics taxonomy: A flow chart displaying the toxicokinetics taxonomy**.

The third sub-taxonomy focuses on toxicokinetics, shown in Figure 5, specifying the different parts of this process. It consists of four main classes: "absorption, uptake, distribution, excretion", "bioaccumulation/lipophility", "metabolism", and "toxicokinetic modelling". For example, the class "metabolism" gives information about the distribution of the chemical in the body, e.g. "biodegradation", "metabolic enzymes", "biotransformation".

The resulting taxonomy, consisting of the three sub-taxonomies, includes 48 classes in total. Table 6 shows the total number of abstracts and annotated keywords belonging to each class. It shows that 82.4% of the annotated abstracts include keywords belonging to the "carcinogenic activity" sub-taxonomy, and 50.3% and 28.1% belonging to the "MOA" and "toxicokinetics" sub-taxonomies, respectively. As we go into the deeper levels of the taxonomy, the number of abstracts associated with individual classes gets increasingly small.

**Table 6:** Number of abstracts, keywords and FScore per class

**Class**	**Abstracts**	**Keywords**	**F-Measure**
Carcinogenic activity	1023	1157	92.8
Human study/epidemiology	190 (171)	44	77.7
Tumor related	39	28	56.3
Morphological effect on tissue/organ	2	1	
Biochemical/cellbiological effects	2	3	
Biomarkers	35	14	68.4
Polymorphism	37	32	79.5
Animal study	629 (546)	46	80.2
Study length	156 (3)	3	
2-year cancer bioassay	14	9	
Short and medium	143	110	45.9
Tumors	186	73	74.3
Preneoplastic lesions	150	121	81.2
Morphological effect on tissue/organ	60	50	46.3
Biochemical/cellbiological effects	135	198	52.1
Biomarker	6	3	
Type of animal	452 (388)	166	70.5
Genetically modified animals	73	76	73.5
Cell experiments	319 (313)	28	78.5
Biochemical/cellbiological effects	100	128	58.7
Subcellular systems	2	2	
Study on microorganisms	44	22	85.2
Mode of Action	653	316	85.5
Genotoxic	426 (72)	16	89.1
Strand breaks	32	12	77.4
Adducts	174	11	89.8
Chromosomal change	84 (36)	23	68.2
Micronucleus	47	5	85.9
Chromosomal aberration	35	10	68.2
Mutations	145	38	85.4
Other dna mods	100	52	62.0
Non-genotoxic	324 (8)	4	76.3
Reactive oxygen species	54	26	70.5
Cytoxicity	50	7	62.0
DNA repair	29	8	64.2
Hormonal receptor	47	30	61.6
Effects on cell proliferation	113	30	69.6
Effects on cell death	110	10	83.3
Transcriptional, translational, posttranslational modifications	27	22	61.2
Peroxisome proliferation	3	2	
Inflammation	15	10	
Toxicokinetics	365	269	77.7
Absorption, uptake, distribution, excretion	117	45	69.8
Bioaccumulation/Lipophility	0	0	
Metabolism	275 (152)	36	76.4
Activation or deactivation	191	161	74.8
Reactive oxygen species	7	6	
Toxicokinetic modeling	31	21	84.6

The first column shows the name of a class in the taxonomy. The second column shows the total number abstracts classified in the class (or its sub-classes). The value in brackets is the number of abstracts classified in the class without taking the sub-classes into account. The third column shows the total number of unique keyword annotations for each class. The count does not include the annotations for sub-classes, except for the three top level classes where the number of all keywords (also those of sub-classes) are included.

We conducted an inter-annotator agreement test to measure the agreement with which the experts assigned abstracts to classes via keyword annotation. For each of the eight chemicals (see Table 2), 10 abstracts from 15 journals listed in Table 1 were randomly retrieved from PubMed by using the chemical name as the search term. Two experts performed both relevance and keyword annotation using the final taxonomy in the annotation tool, and we investigated the agreement with which they associated the same abstracts with the same classes. We used the Kappa measure introduced earlier in section 2.3.1.

Because the classes are hierarchically organized, disagreement on a child class may still mean agreement on the parent class. For example, annotator 1 (A1) may select "type of animal" while annotator 2 (A2) may select "animal study". Although these classes are not identical they are related as "animal study" is the parent class of "type of animal", and therefore there is an implicit agreement on the class "animal study". We calculated the agreement based on both explicit agreement and implicit (parent) agreement. However, the same parent agreement was counted only once for each abstract. For example, if A1 selected "animal study" and A2 "type of animal" and "study length", the two implicit parent agreements on "animal study" were counted as one agreement only.

Table 7 shows the number of annotated unique keywords along with the agreement statistics. We can see that one annotator (A1) produced significantly more annotations than the other one (A2): 78 in total. This applies to the "carcinogenic activity" sub-taxonomy and in particular to "toxicokinetics" for which A1 produced 1.67 times more annotations than A2. With the MOA taxonomy an opposite trend can be seen: A2's proportion is significantly bigger than that of A1.

**Table 7:** The distribution of the annotations and the statistics of agreement

	**A1**	**A2**	**Agreement**	**Disagreement**
Carcinogenic activity	281 (0.55)	217 (0.50)	194 (0.78)	55 (0.22)
Mode of Action	158 (0.31)	172 (0.40)	129 (0.78)	36 (0.22)
Toxicokinetics	75 (0.15)	45 (0.10)	37 (0.62)	23 (0.38)
Irrelevant	0	2	0	2
Total	514	436	360 (0.76)	116 (0.24)

The columns A1 and A2 correspond to the annotators 1 and 2, respectively. The values shown are the number of annotations by the annotator. The last two columns show the statistics of agreement and disagreement. Rows 2-4 show the results for the three sub-taxonomies and the last row indicates the number of irrelevant abstracts among the relevant ones.

The average agreement between the annotators is the highest with "carcinogenic activity" and "MOA" at 78%. With "toxicokinetics" it is significantly lower (62%). The overall agreement is 76%. These results are good, particularly considering that the annotation was done using a relatively high number of classes and the chance agreement is low at 1.5%.

We conducted error analysis of the annotations the annotators disagreed on. The main source of disagreement was the different annotation style of the two annotators. A1 annotated as many words as possible, aiming for a maximum number of taxonomy classes per abstract. A2 annotated just one or a few words that classify the abstract as precisely as possible. In other words, A1 focussed on word-level annotation while A2 focussed on document level annotation (even when annotating keywords). Both approaches are plausible (given the annotation guidelines) and both resulted in annotations useful for CRA. The error analysis revealed that some classes are not specific enough to yield unique distinctions and as a consequence, some keywords are even assigned to different sub-taxonomies. For example, A1 typically assigned protein changes to "biochemical effects" in the "carcinogenic activity" sub-taxonomy, while A2 assigned them to e.g. "post-translational modifications" in the "MOA" sub-taxonomy. Both annotations are plausible given the current model, but future work should focus on refining the taxonomy further to obtain clearer distinctions.

### Automatic classification

We evaluated the automatic classification against the expert annotated CRA corpus. We used the standard evaluation measures of recall (R), precision (P) and F measure (F) to evaluate the rate with which the classification assigned abstracts to the correct taxonomy classes. These measures are defined as follows:



where *P*_+_/*N*: positive/negative population; TP: true positive; FN: false negative, and FP: false positive. Our random baseline is .

#### Document preprocessing

We first evaluated the *BOW *preprocessing technique with and without the use of (i) the Porter stemmer [29], (ii) TFIDF, (iii) stop word removal, and (iv) their combinations. The evaluation was done in the context of binary relevance classification of abstracts (not in the context of the main taxonomic classification task to avoid overfitting preprocessing techniques to the taxonomy). Only (iii) improved the performance of all the classifiers (data not shown here) and was thus adopted for the main experiments. The poor performance of (i) demonstrates that a standard stemmer is not optimal for biomedical data. As highlighted by Han *et al*. and Wang *et al*. [31,32], semantically related biological terms sharing the same stem are not always reducible to the stem form.

#### Feature selection

We evaluated the feature selection methods with two taxonomy classes: the most balanced class 'animal study' (positive/negative 1:1.4) and an imbalanced class 'adducts' (positive/negative 1:6.5). *IG *was used for the fixed *N *setting and *fscore *for the *dynamic N *setting. Each combination of classifiers (*NMB/CNB/SVM*), document representations (*BOW, BOS*) and settings for *N *(dynamic,...,83098) was evaluated. The results showed that the *dynamic *setting yields consistent improvement for all the setups (although the impact on *SVM'*s is not big) and that the optimal *N *varies by the data and the classifier (the data not shown). Thus, we used the *dynamic *feature selection in the taxonomic classification.

#### Taxonomic classification

We ran two sets of experiments on the corpus, using 1) *BOW *and 2) *BOS *for feature extraction. Without feature selection, *BOW *had c. 9000 features and *BOS *c. 83000. Features were selected using *fscore*. For each class with more than 20 abstracts (37 in total)^5^, three "one against other" classifiers (*NMB*, *CNB *and *L-SVM*) were trained and tested using a standard 10-fold cross validation.

Table 8 shows the average performance across the whole taxonomy. The performance of *BOS *is better than that of *BOW *according to all the three measures. On average, *BOS *outperforms *BOW *by 4% in P and F, and 3% in R. *SVM *yields the best overall P and F (*0.71 *and *0.74*) with *BOS*. Surprisingly, *NMB *outperforms *CNB *with all the settings. *NMB *yields the best overall R with *BOS *(0.82) but its P is notably lower than that of *SVM*.

**Table 8:** Performance of classifiers with BOS and BOW

**Method**	**Feature Set**	**P**	**R**	**F**
NMB	BOW	0.59	0.75	0.66
NMB	BOS	0.62	0.82	0.70

CNB	BOW	0.52	0.74	0.60
CNB	BOS	0.57	0.76	0.64

SVM	BOW	0.68	0.76	0.71
SVM	BOS	0.71	0.77	0.74

Table 9 shows the average P, R and F for the three sub-taxonomies using the best performing feature set *BOS *with the three classifiers. "Carcinogenic activity" (*CA*) has the best F (0.93). Its positive population is the highest (positive/negative: 5:1). "Toxicokinetics" (*TOX*) with a lower positive population (1:2.6) has still good F (0.78). In these results, R and P are balanced with an average difference of 0.06.

**Table 9:** Results for the three sub-taxonomies

**Class**	**Method**	**P**	**R**	**F**
CA	NMB	0.94	0.89	0.91
CA	CNB	0.92	0.94	0.93
CA	SVM	0.93	0.93	0.93

MOA	NMB	0.88	0.81	0.84
MOA	CNB	0.84	0.82	0.83
MOA	SVM	0.92	0.80	0.86

TOX	NMB	0.66	0.83	0.74
TOX	CNB	0.70	0.80	0.75
TOX	SVM	0.76	0.79	0.78

Table 10 shows the distribution of F across the taxonomy for different frequency ranges. There is a clear correlation between frequency and performance: the average F decreases with descending frequency range, revealing increased classification difficulty. Classes with more than 300 abstracts have the highest average F (0.80 with standard deviation (*SD*) 0.08). Classes with 20-100 abstracts have the average F 0.68 (*SD *0.11), which is lower but still fairly good. No class has F lower than 0.46, which is much higher than the average random baseline of 0.11.

**Table 10:** Mean F and random baseline for taxonomic classes in three frequency ranges

**No. of abstracts(f)**	**Classes**	**F**	**Random**
*f *> 300	9	0.80	0.38
100 <*f *≤ 300	12	0.73	0.13
20 <*f *≤ 100	16	0.68	0.04

In sum, these experiments demonstrate that the taxonomy we have created is machine learnable with high accuracy for the classes for which sufficient corpus data is available.

### User Test

A small user test was finally carried out to investigate the practical usefulness of the automatic classification in a near real-world CRA scenario. In this test, the best classifier (*L-SVM+BOS*) trained on the CRA corpus (as explained in the above section) was applied to the PubMed abstract data of five unseen chemicals which represent the same genotoxic (geno) and non-genotoxic (non) MOAs (see Table 11). The abstracts were downloaded from the set of 15 journals listed in Table 1. As with the CRA corpus, only abstracts from years 1998-2008 were included.

**Table 11:** Unseen chemicals and the results of the user test

**Name**	**MOA**	**Σ**	**P**	**Class**	**P**
Aflatoxin B1	geno	189	0.95	CA	0.94
Benzene	geno	461	0.99	MOA	0.95
PCB	non	761	0.89	TOX	0.99
Tamoxifen	non	382	0.96		
TCDD	non	641	0.96		

The results were displayed to one of our experts in a web interface. The expert was invited to imagine that she had submitted a query to a TM system, the system had classified each abstract of each chemical to relevant taxonomy class(es), and the task is to judge whether the proposed classification is correct. The top 500 *BOS *features per class were shown to the expert to aid the judgement.

The results were evaluated using precision (P) (recall could not be calculated as not all of the positive polulation was known). Table 11 shows the average P per each (i) chemical and (ii) sub-taxonomy. The results are impressive: the only chemical with P lower than 0.90 is polychlorinated biphenyls (PCB). As PCB has a well-known neuro-behavioural effect, the data includes many abstracts irrelevant for CRA. The good performance can be observed across the whole taxonomy: *TOX *has the best P (0.99), and *CA *and *MOA *have 0.94 and 0.95 P, respectively. Most errors are due to the lack of training data for low frequency classes. For example, the CRA corpus has only 27 abstracts in "DNA repair (damage)" class, while the data for new chemicals have many.

The expert found this evaluation easy to conduct. She felt that if such an automatic classification system was available to support real-world CRA, it could significantly increase the productivity and also lead to more consistent and thorough CRA since manual gathering of such a wide range of scientific evidence from abstracts is very difficult. This result is encouraging. Larger tests using several experts are required to investigate the full performance of automatic classification on unseen corpus data. We plan to conduct such tests after we have extended the initial taxonomy further to cover additional, finer-grained MOA types (as described in the following section).

## Discussion and Conclusion

The results of our inter-annotator agreement tests, automatic classification experiments and the user test all demonstrate that the taxonomy created by risk assessors is accurate, well-defined, and can be useful in practice. This is particularly encouraging considering that the taxonomy is based on expert annotation of biomedical texts.

The annotation of biomedical corpora is a challenging task [24]. It is also an important task since most current TM approaches rely on annotated corpora and are therefore dependent on the quality of these resources. Various annotation schemes have been proposed which involve either linguistic or expert annotation. As highlighted by Kim *et al *[46], expert annotation is more challenging and more prone to inter-annotator disagreement than better-constrained linguistic annotation. We believe that we obtained promising results regardless of this because our interdisciplinary team included also risk assessors: we developed an annotation approach which imitates their current practices as closely as possible and involves gathering information specifically for their needs. Like the recent user-centered annotation scheme of Wilbur *et al*. [6] it can support the classification of biomedical literature along various qualitative dimensions. However, with the focus on CRA, our scheme is specifically aimed at classifying cancer related evidence. The latter can be useful for risk assessment and for e.g. researchers working on cancer research. A number of works have been reported on disease- and drug-related TM which have involved similar knowledge acquisition and classification efforts as our work, e.g. [47-49] among others. Although some prior work has been done on cancer-related TM, the work conducted so far has focussed on tasks such as building cancer-related databases (e.g. a cancer methylation database [50]), detecting associations between cancer and specific genes or proteins [51], classifying abstracts based on the type of cancer they focus on (e.g. the breast vs. lung cancer) [52], and mining clinical records for cancer diagnosis [53]. No prior work has been done (to the best of our knowledge) on specifying such a wide range of cancer-related evidence or developing TM for risk assessment of potentially carcinogenic substances.

The work we have presented in this paper constitutes the first step towards developing TM for CRA. The taxonomy we have constructed provides the practical means to identify key evidence in CRA literature and to classify this evidence in semantically meaningful classes. The ability to assign journals, abstracts, and experimental results in the taxonomy can help risk assessors to (i) keep track of the evidence covered/not covered and (ii) detect important statistical tendencies in the CRA literature, e.g. that most of the scientific data provides evidence for some specific MOA type.

In the future, we plan to develop and extend the taxonomy further. Although our results show that the current taxonomy provides a good basis for the classification of CRA literature, it is not comprehensive: more data is required especially for low frequency classes, and the taxonomy needs to be adapted and extended to cover more specific MOA types (e.g. further subtypes of non-genotoxic chemicals) and novel findings.

The taxonomy can be extended using manual annotation, by supplementing it with additional information in knowledge resources and/or using automatic methods. A number of extensive knowledge resources have been built for biomedicine which also enable classifying concepts in biomedical texts in semantically coherent classes. The most prominent of these are widely used to support biomedical text mining tasks, e.g. the Medical Subject Headings (MESH) ontology [54] and the Unified Medical Language System (UMLS) knowledge sources (Metathesaurus, the Semantic Network, and the Specialist Lexicon) [55]. Although these general resources lack a number of concepts important for CRA (e.g. MOA), cover a large number of concepts irrelevant for CRA, and organize many similar concepts differently (e.g. do not organize scientific studies according to their length), some information provided in them could further support CRA and would therefore be worth exploring.

We performed a small experiment to investigate the usefulness of MeSH for supplementing our current classification. MeSH terms were first retrieved for each abstract using EFetch [56] and then appended to the *BOS *feature vector. The best features were selected using *fscore *and classified using *L-SVM*. The figures included in Table 12 show that the classification improved significantly for 43% of the classes, the majority of which are low in frequency. Although this demonstrates the potential usefulness of additional manually built resources, given the rapidly evolving nature of CRA data, the best approach long term is to develop technology for automatic updating of the taxonomy from literature. Given the basic resources we have constructed and presented in this paper, the development of such technology is now realistic and can be done using unsupervised or semi-supervised machine learning techniques, e.g. [1,57].

**Table 12:** F gain(Δ_*F*_) of MeSH compared to BOS

		**Distribution of abstracts per class freq. range**		
		
**Change in F**	**Σ Classes**	**20-100**	**100 - 200**	**200 - 1100**
Δ_*F *_> 1%	16 (43%)	75%	33%	8%
|Δ_*F*_| ≤ 1%	15 (41%)	6%	44%	75%
Δ_*F *_< -1%	6 (16%)	19%	33%	17%

Our simple automatic classification method could be improved in various ways. An obvious way to improve it is to extend the shallow feature set with more sophisticated features extracted using NLP tools that have been tuned for biomedical texts, such as taggers and parsers, e.g. [58], named entity recognizers, e.g. [59], and exploiting lexical resources such as the BioLexion [60].

Given the basic resources described in this paper and the proposed extensions, our long term goal is to develop a TM tool to support the entire CRA workflow. Our initial study (the interviews conducted with risk assessors, see the Introduction section) revealed that a tool capable of identifying, ranking and classifying articles based on the evidence they contain, displaying the results to experts, and assisting also in the subsequent steps of CRA would be welcome. Such a tool could significantly increase the productivity and consistency of CRA and enable risk assessors to concentrate on what they are best at: the expert judgement. It could also, as a side-effect, keep track of the CRA process, providing the practical means to address one of the biggest current problems in CRA: the need for improved consistency and transparency of risk assessments [23].

Such a tool should be developed in close collaboration with risk assessors. The interface should be easy to use, interactive, support search in a graphical manner, include a helpful statistical summariser, and enable the storage of interesting results in specific collections. It should provide online access to CRA guidelines and resources, and be sufficiently flexible to permit specific searchers in selected repositories of literature. Ideally, it should be designed in a way that it facilitates also the subsequent steps of CRA, e.g. the analysis of the retrieved data, the discussion among the CRA team and the subsequent generation of the CRA report.

A number of tools have recently been built to assist other critical activities in biomedicine (e.g. literature curation for genetics, literature search in plant related literature) [10-13]. A few of them have also been evaluated for their practical usefulness in a real-world scenario [8,9]. Such tools and evaluations act as an important proof of concept for biomedical TM as well as enable improving existing technology according to the needs of practical applications.

## Appendix

### Appendix 1 Sentences from abstracts relevant for CRA

Keywords representing evidence for relevance are indicated in bold font

Although A:T to T:A **transversions **were the major form of mutation observed following treatment with each of the three stereoisomers (35-40%), S, S-DEB induced higher numbers of G:C to A:T **transitions**, whereas R, R-DEB treatment resulted in a greater frequency of G:C to T:A transversions.

Measurements of **Hprt mutant frequencies **(via the T cell cloning assay) showed that repeated exposures to 18 and 36 ppm BD-diol were significantly **mutagenic **in mice and rats.

All three BD exposure indices were associated positively with **leukemia**.

Assays for the N, N-(2,3-dihydroxy-1,4-butadyl) valine (pyr-Val) hemoglobin (Hb) **adduct**, which is specific for the highly **genotoxic **1,2,3,4-diepoxybutane (DEB) metabolite of BD, have been conducted on blood samples from all participants in this second Czech study.

Thus, in terms of mutagenic efficiency, stereochemical configurations of EB and DEB are not likely to play a significant role in the **mutagenicity **and **carcinogenicity **of BD.

Thus, in mouse liver, the trihalomethanes administered by gavage enhanced cell proliferation and decreased the **methylation **of the **c-myc **gene, consistent with their **carcinogenic **activity.

The data support the hypothesis that PB promotes **neoplastic development **through a reduction in the incidence of **cell death**.

### Appendix 2 Example sentences containing evidence for irrelevance

Example sentences containing information (indicated in bold font) which suggests that the abstract is irrelevant for CRA

Exposure to 600 ppm styrene caused a 3 dB **hearing loss **only at the highest test frequency (8 kHz).

**Asthma **symptom severity was regressed on pollutants using generalized estimating equations, and peak expiratory flow (PEF) was regressed on pollutants using mixed models.

Collectively, our results indicated that chloroform directly and concentration-dependently provoked **muscle contraction **in swine **tracheal smooth muscle**.

These results demonstrate that BaP/DMBA causes a loss of bone mass and **bone strength**, possibly through an increase in bone turnover.

### Appendix 3 The 16 additional journals used for inter-annotator agreement test

Journals

Journal of Biological Chemistry

PNAS (Proc Natl Acad Sci)

Pancreas

Bone

Endocrinology

Regulatory Toxicology and Pharmacology

Epidemiology

Blood

Toxicologic Pathology

International Journal of Toxicology

Risk Analysis

Cell Death and Differentiation

American Journal of Epidemiology

American Journal of Industrial Medicine

Toxicology

European Journal of Pharmacology

### Appendix 4 Footnotes

1. Institute of Environmental Medicine at Karolinska Institutet, Swedish Chemical Inspectorate, Scientific Committee on Occupational Exposure Limits (EU), Swedish Criteria Group.

2. Chemicals acting by a genotoxic MOA interact with DNA, while chemicals acting by a non-genotoxic MOA induce cancer without interfering directly with DNA.

3. Minus 2 because of space characters.

4. Since our third expert was not available during the inter-annotator agreement tests, the tests were conducted using two experts only.

5. The classes with less than 20 abstracts may have less than 2 positive abstracts in each fold of 10 fold CV, which is not representative of the class population.

## Authors' contributions

All four authors participated equally in the work reported in this paper. AK wrote most of the paper and together with US designed, supervised and coordinated the project. IS conducted the annotation work, designed the taxonomies, and conducted the inter-annotator agreement tests and the user test with the assistance of US. LS developed the annotation tool, constructed the CRA corpus, implemented the automatic classification approach and performed the evaluation of these resources with the assistance of AK. All authors have read and accepted the final manuscript.
